# Long-term Safety and Effectiveness of Rezafungin Treatment in Candidemia and Invasive Candidiasis: Results From an Early Access Program in Italy and Germany

**DOI:** 10.1093/ofid/ofaf034

**Published:** 2025-03-19

**Authors:** Filippo Trapani, Giulio Viceconte, Valentina Morena, Giusy Tiseo, Giovanni Mori, Britta Kölking, Elham Khatamzas

**Affiliations:** Infectious Disease Unit, Department of Oncology and Hematology, Guglielmo da Saliceto Hospital, Piacenza, Italy; Infectious Diseases Unit, Department for Integrated Infectious Risk Management, IRCCS Azienda Ospedaliero-Universitaria di Bologna, Bologna, Italy; Section of Infectious Diseases, Department of Clinical Medicine and Surgery, University of Naples Federico II, Naples, Italy; Infectious Diseases Unit, “A. Manzoni” Hospital, ASST Lecco, Italy; Infectious Diseases Unit, Department of Clinical and Experimental Medicine, Azienda Ospedaliera Universitaria Pisana, University of Pisa, Pisa, Italy; Infectious Diseases Unit, Azienda Provinciale per i Servizi Sanitari (APSS), Trento, Italy; Università Vita-Salute San Raffaele, Milano, Italy; Department of Infectious Diseases and Tropical Medicine, Centre for Infectious Diseases, Heidelberg Hospital, Heidelberg, Germany; Department of Infectious Diseases and Tropical Medicine, Centre for Infectious Diseases, Heidelberg Hospital, Heidelberg, Germany; German Centre for Infection Research (DZIF), Partner site Heidelberg University Hospital, Heidelberg, Germany

**Keywords:** candidemia, early access program, echinocandin, invasive candidiasis, rezafungin

## Abstract

Outcomes are reported for 6 adults receiving rezafungin for chronic, hard-to-treat, invasive candidiasis (including *Candida parapsilosis*) during an early access program. Rezafungin was well tolerated and administered via once-weekly outpatient intravenous infusion for up to 39 weeks during the program, enabling hospital discharge and replacing daily antifungal infusions.

Patients with candidemia and/or invasive candidiasis have high morbidity and mortality risk, and emergence of treatment-resistant *Candida* species (particularly non-*albicans* species) places further pressure on clinicians to identify effective antifungal therapies that extend survival and preserve quality of life (QoL) [1–6]. Guidelines recommend timely diagnosis and early treatment, which includes antifungals with surgical intervention and complete removal of infected foreign material [7, 8]. Patients unable to undergo surgical resection require prolonged (even lifelong) antifungal therapy [7, 8]. Echinocandins are among the recommended first-line treatments in Europe and the United States [7, 8]. Before rezafungin approval, all available echinocandins were administered via daily intravenous (IV) infusion, requiring patients to remain in the hospital or attend daily outpatient parenteral antimicrobial therapy (OPAT) services [9].

Rezafungin is a next-generation, once-weekly echinocandin demonstrating potent in vitro activity against a range of *Candida* species (including some azole-resistant strains) and a prolonged half-life, with low risk of drug–drug interactions [9–15]. Rezafungin is approved in the European Union, United Kingdom, and United States for invasive candidiasis and candidemia treatment in adults [10,16–18]. Clinical studies revealed rezafungin to be effective in treating invasive *Candida* infections, with a similar safety and tolerability profile to caspofungin [19–21]. In the phase 2 STRIVE trial, overall cure rates were 76.1% with once-weekly IV rezafungin (week 1: 400 mg; week 2 onwards: 200 mg) and 67.2% with daily caspofungin (day 1: 70 mg; day 2 onwards: 50 mg), and day 30 all-cause mortality (ACM) was 4.4% (rezafungin) and 13.1% (caspofungin) [19]. The phase 3 ReSTORE trial reflected STRIVE outcomes, with day 14 global cure rates of 59% (rezafungin) and 61% (caspofungin) and day 30 ACM being 24% (rezafungin) and 21% (caspofungin) [20].

Here, we report outcomes from an early access program (EAP), which aimed to provide rezafungin for eligible patients and to expand knowledge regarding rezafungin safety and tolerability. Outcomes have already been reported for some individual EAP patients (Cases 1–3) [22–24]. The current paper therefore summarizes data for the full program cohort, with unpublished cases highlighted in more detail (Cases 4–6).

## METHODS

The rezafungin EAP complied with the Declaration of Helsinki and local regulations/ethics committee requirements. The Italian Medicines Agency (AIFA) and German Federal Institute for Drugs and Medical Devices (BfArM) approved the protocol. Patients gave written informed consent before participation.

Patients in Italy and Germany with candidemia/invasive candidiasis were included in the EAP between November 10, 2022 (Italy), or December 15, 2022 (Germany), and February 17, 2024. Inclusion was contingent on the presence of ≥1 eligibility criterion: ineligible for currently available therapies (impaired liver function, difficult venous approaches, major drug interaction risk); eligible for discharge but needing daily IV infusions; OPAT required; receiving critical care (with fluid overload); azole-resistant and echinocandin-susceptible infection; infusion site reactions (fewer infusions preferable); long-term treatment (>4 weeks) required. Patients in Germany were ineligible for ongoing rezafungin trials, with unsatisfactory outcomes on existing antifungal regimens. Pregnant/breastfeeding women were not included.

### Study Treatment

Eligible patients received a single 400 mg rezafungin dose on day 1, followed by optional dosing (200 mg) on day 8 and once-weekly thereafter. Treatment duration was based on clinical and microbiological response. Rezafungin IV infusions were 60 (±10) minutes, with the option to increase infusion time up to 180 minutes.

### Study Assessments and End Points

Efficacy assessments (performed on day 15, day 29, and end of therapy) included *Candida* pathogen detection, concomitant antifungal medications, re-hospitalization due to *Candida* infection, and incidence and cause of death. Length of rezafungin treatment and reasons for discontinuation were recorded. Adverse events (AEs) were reported throughout.

## RESULTS

### Patient Demographics and Characteristics

Ten patients provisionally enrolled in the EAP, and 6 received ≥1 rezafungin dose (5 in Italy; 1 in Germany) (Table 1). Of the 4 who did not receive rezafungin during the EAP, 1 was treated with rezafungin as part of a named-patient program (due to batch availability; Italy), 1 died before the start of treatment (Italy), 1 did not start rezafungin due to organizational issues (Italy), and 1 was discharged early from the hospital (Germany). Five rezafungin recipients were male, and the mean age (range) was 65.5 (45–74) years. Diagnoses included foreign body infections (spondylodiscitis involving nonremovable hardware, prosthetic valve endocarditis, vascular graft infection), candidemia, native valve endocarditis, and intra-abdominal candidiasis. Two patients had been treated in the intensive care unit before the EAP. None of the patients received mechanical ventilation. *Candida* infection risk factors included renal disease, peripherally inserted central catheter, major surgery, and diabetes mellitus. Detected *Candida* species comprised *C. parapsilosis*, *C. tropicalis, C. glabrata,* and *C. albicans*.

**Table 1.** Baseline Patient Demographics and Characteristics

	Case 1	Case 2	Case 3	Case 4	Case 5	Case 6
Country	Italy	Italy	Italy	Italy	Italy	Germany
Sex	Male	Male	Female	Male	Male	Male
Age, y	69	68	70	66	74	46
BMI, kg/m^2^	26.0	22.9	17.6	27.7	25.3	22.0
Rezafungin therapy duration	36 wk^b^	26 wk	8 wk	12 wk^b^	5 wk	39 wk^b^
*Candida* risk	Major surgery, total parenteral nutrition, catheter-related bloodstream infection	Central catheter, total parenteral nutrition	Corticosteroid use, diabetes mellitus	…	Central venous catheter, major surgery	Diabetes mellitus type 1, major surgery, end-stage renal disease, hemodialysis
Care setting	Outpatient	Outpatient	General ward (discharge allowed)	General ward	Outpatient	Outpatient
Diagnosis	Endocarditis, prosthetic valve infection, TEVAR, liver and hepatic embolisms	Spondylodiscitis	Native valve (aortic) endocarditis	*Candida* endocarditis on aortic prosthetic valve and infection of ascending aortic prosthesis, endophthalmitis (receiving fluconazole)	Candidemia, prosthetic valve endocarditis	Spondylodiscitis
Foreign body infections	Yes	No	No	Yes	Yes	No
Reason for rezafungin use	Outpatient IV antifungal treatment, azole resistance	Outpatient IV antifungal treatment, reduced susceptibility to azoles, intolerance to azoles and amphotericin	Outpatient IV antifungal treatment, intermediate susceptibility to fluconazole (MIC 8 mg/L), drug interaction risk with fluconazole	Outpatient IV antifungal treatment	Outpatient IV antifungal treatment	Outpatient IV antifungal treatment, long-term treatment required
*Candida* species detected	*C. tropicalis* (azole resistant)	*C. parapsilosis* ^a^	*C. glabrata*	*C. parapsilosis*	*C. parapsilosis*	*C. albicans, C. glabrata*
Anidulafungin MIC Level, mg/L	0.03	1.0	0.03	0.125	N/A	0.016
Caspofungin MIC level, mg/L	0.016	0.5	0.25	0.125	N/A	N/A
Micafungin MIC level, mg/L	0.03	2.0	0.16	0.125	N/A	0.003
Previous antifungal therapy	Fluconazole, liposomal amphotericin B, caspofungin, isavuconazole	Anidulafungin, voriconazole, liposomal amphotericin B	Anidulafungin	Fluconazole, caspofungin, liposomal amphotericin B	Fluconazole, anidulafungin	Caspofungin

Rezafungin susceptibility for detected isolates was inferred based on MICs for anidulafungin and micafungin.

Abbreviations: BMI, body mass index; EAP, early access program; IV, intravenous; KDIGO, Kidney Disease Improving Global Outcomes; MIC, minimum inhibitory concentration; N/A, not available; TEVAR, Thoracic EndoVascular Aneurysm Repair.

^a^Reduced susceptibility to azoles.

^b^Patients continued rezafungin therapy after the end of the EAP study period. Case 1: up to 9 months; Case 4: up to 12 months; Case 6: treatment ongoing at 19 months.

Six patients received antifungals (≥2 weeks) before rezafungin, comprising caspofungin, voriconazole, liposomal amphotericin B, fluconazole, isavuconazole, or anidulafungin. Case 1 continued isavuconazole with rezafungin throughout the EAP. Three patients had infections demonstrating reduced azole susceptibility, including 1 with intolerance to azoles/amphotericin B and 1 in which fluconazole represented a drug interaction risk. Rezafungin was selected to facilitate hospital discharge and outpatient administration.

### Duration and Outcomes of Therapy

The 6 rezafungin recipients were treated for 5–39 weeks during the program. Blood cultures measured for Case 3 (day 15) and Case 5 (day 29 and end of therapy) remained negative. Four patients received rezafungin until the end of the program. Cases 1 and 4 continued rezafungin after the EAP, up to 10 and 12 months, respectively. Case 6 also continued, with therapy ongoing at 19 months.

Overall, 3 patients experienced AEs during the EAP study period, all of which were severe (grades 3, 4, and 5) and led to hospitalization. Two patients discontinued rezafungin before the end of the program (Cases 3 and 5). Case 3 died of probable urosepsis (*Klebsiella pneumoniae*) unrelated to rezafungin (after 8 weeks of rezafungin treatment) with consistent clinical and laboratory response until the urosepsis. Rezafungin had also been discontinued for 1 week at the time of death. Case 5 was unable/unwilling to continue rezafungin after 5 weeks following cardiac failure (not related to rezafungin treatment or *Candida* endocarditis), which he recovered from. Case 2 experienced radiological worsening during the early phase of the rezafungin EAP (unrelated to rezafungin) and fully recovered during the program without changing antifungal therapy. While Case 1 continued rezafungin with isavuconazole beyond the end of the EAP (up to 10 months), the patient discontinued isavuconazole (after 11 months) due to suspected liver toxicity. At this stage, his positron emission tomography–computed tomography scan was negative and showed no metabolic activity around the thoracic endovascular aneurysm repair (TEVAR) and aortic valve. Rezafungin was continued, but the patient died 2 months after isavuconazole interruption, and blood cultures were positive for *Candida tropicalis* (susceptible to echinocandins, minimum inhibitory concentration [MIC] 0.125). A brain magnetic resonance image showed 2 lesions suggestive for septic embolism. Neither AEs nor liver toxicity were associated with rezafungin.

As highlighted in the introduction, outcomes have already been published in detail for Cases 1–3 [22–24]. Details relating to Cases 4–6 are summarized below.

Case 4 was a 66-year-old male (Italy). He had undergone aortic valve and ascending aortic replacement and developed candidemia complicated by endocarditis, infection of the ascending aortic prosthesis, endophthalmitis, and cerebral ischemia while in the hospital. *C. parapsilosis* was detected at diagnosis, and he received fluconazole, caspofungin, and liposomal amphotericin B before rezafungin initiation. Negative blood cultures and negative C-reactive protein values were demonstrated at EAP day 15. He had received rezafungin plus fluconazole for 12 weeks at the end of the EAP and continued therapy up to 12 months, after which time he received only fluconazole.

Case 5 was a 74-year-old male (Italy) with prosthetic valve endocarditis. *C. parapsilosis* was isolated at diagnosis. Despite receiving several antifungal therapies (alone and in combination), he experienced reoccurrence of candidemia and endocarditis. Anidulafungin was given after a new surgical repair, but treatment was switched to rezafungin with the aim of improving QoL and preventing further reoccurrence of infection. Rezafungin was stopped after 5 weeks as the patient had cardiac failure (unrelated to rezafungin) and was unwilling to continue treatment.

**Figure 1.**
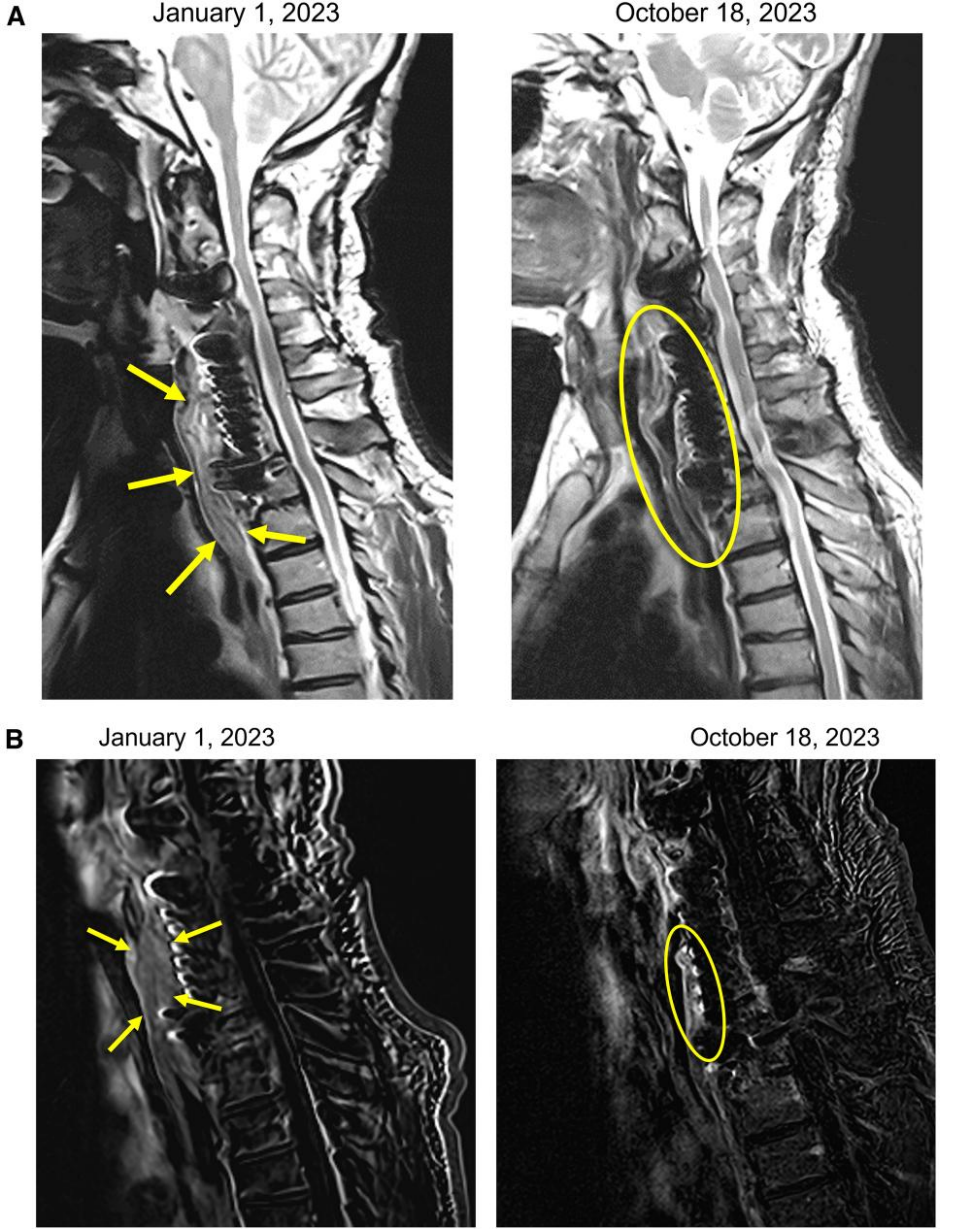
Computed tomography scan images for Case 6 at diagnosis and at 8 months after starting treatment with echinocandin including rezafungin, showing regression of prevertebral soft tissue swelling. The patient required lifelong antifungal therapy and continued rezafungin treatment after the early access program period (ongoing at 19 months). *A*, T2 sagittal image of the cervical spine. *B*, Contrast media subtraction images.

Case 6 was a 46-year-old male (Germany) with diabetes mellitus and end-stage renal disease who had undergone prior major spinal fixation surgery and developed hardware-associated cervical spondylodiscitis (Figure 1). The patient had undergone multiple cervical spinal operations during the preceding 12 years, with cage and osteosynthesis insertions and revisions. *C. albicans* and *C. glabrata* were detected at diagnosis. Before rezafungin, caspofungin and fluconazole were given on the general ward. Fluconazole was ceased due to significant liver function test abnormalities. Rezafungin was initiated to enable long-term treatment in the outpatient dialysis setting. He had received rezafungin for 39 weeks at the end of the EAP and continued treatment after the program (ongoing at 19 months).

## DISCUSSION

The EAP enabled patients with invasive candidiasis/candidemia, aged 46–74 years, to receive rezafungin therapy. Most had received prior azole, echinocandin, or polyene antifungal therapy. Negative blood cultures reported during the EAP indicated antifungal efficacy with rezafungin against predominantly non-*albicans* species, including some azole-resistant (or limited susceptibility) strains, and treatment was well tolerated [22,23]. While the patient numbers included in the program were small and the population was relatively heterogeneous compared with large-scale randomized trials, the reported outcomes provide insights regarding the use of rezafungin in clinical practice and the types of patients who may benefit from this kind of antifungal therapy.

Rezafungin susceptibility for detected isolates was inferred, based upon MICs for anidulafungin and micafungin due to previously demonstrated similarities across the treatment class [25]. While susceptibility of the detected isolates was therefore likely to be similar to that of other echinocandins, the prolonged elimination half-life of rezafungin enabled once-weekly outpatient dosing, rather than daily IV infusions [9–15]. Outpatient rezafungin administration allowed patients to remain at home between visits, where they may have felt more comfortable and had improved QoL. Economic modeling, based on the rezafungin STRIVE study, has suggested this approach to be cost-saving due to reduced length of hospital stay, and avoidance of daily infusions may help lower costs of potential line-related complications [26]. Unfortunately, the study protocol did not include collection of QoL information (eg, via a validated questionnaire/tool), and a robust economic evaluation of the program outcomes was not possible due to the small patient population and complexities associated with conducting the EAP in a range of hospital settings across Italy and Germany. However, collection of data regarding these aspects of patient care would be of value for future programs and would help to inform decisions regarding the most appropriate setting of treatment for patients requiring long-term antifungal therapy.

Of the 6 rezafungin recipients, 4 remained on treatment until the end of the EAP, receiving therapy for up to 39 weeks. Cases 1, 4, and 6 continued rezafungin treatment after the EAP, with Case 4 ceasing treatment at 12 months (and continuing with fluconazole therapy) and Case 6 still receiving treatment at 19 months. Case 1 died after 10 months of rezafungin plus isavuconazole treatment. EAP outcomes therefore suggest that rezafungin may represent an important option for patients requiring prolonged antifungal therapy, such as those with endocarditis included in the program [22,27–29].

Common issues with antifungal treatments include poor tolerability, hepatotoxicity, drug–drug interactions, and cardiotoxicity, including QT interval prolongation [30]. Patients in the EAP had also experienced hypokalemia due to prior azole treatment. As highlighted in the results for Case 1, isavuconazole was paused due to liver toxicity, and the patient subsequently died with signs of septic embolism. A possible explanation may be that the patient had an undiagnosed central nervous system (CNS) involvement that was controlled by isavuconazole due to better therapeutic concentrations in the CNS than rezafungin. Rezafungin was well tolerated, with no treatment-related AEs or indicators of liver toxicity reported during the EAP. Previous studies showed that no dose adjustment was required for rezafungin in the elderly or patients with renal impairment, and the EAP results provide reassuring evidence of tolerability in these groups [16–18]. Five rezafungin recipients were aged >65 years, and Case 6 had end-stage renal failure before the EAP, yet they remained on rezafungin for the longest period during the program.

## CONCLUSIONS

Patients from Italy and Germany with chronic invasive *Candida* infections received rezafungin in the outpatient setting for up to 39 weeks during the EAP, with some continuing long-term therapy after the program ended. The EAP provided an opportunity for patients with limited treatment options and prolonged (potentially lifelong) antifungal requirements to receive rezafungin. Outpatient administration supported patient QoL, and rezafungin was generally effective and well tolerated.
